# Immersive virtual reality gameplay detects visuospatial atypicality, including unilateral spatial neglect, following brain injury: a pilot study

**DOI:** 10.1186/s12984-023-01283-9

**Published:** 2023-11-23

**Authors:** David R. Painter, Michael F. Norwood, Chelsea H. Marsh, Trevor Hine, Daniel Harvie, Marilia Libera, Julie Bernhardt, Leslie Gan, Heidi Zeeman

**Affiliations:** 1 The Hopkins Centre, Menzies Health Institute Queensland, Griffith University, 170 Kessels Rd, Nathan, QLD 4111 Australia; 2 School of Applied Psychology, Griffith University, Gold Coast, QLD Australia; 3 School of Applied Psychology, Griffith University, Mount Gravatt, QLD Australia; 4 Allied Health and Human Performance, Innovation, Implementation and Clinical Translation in Health (IIMPACT in Health), University South Australia, Adelaide, SA Australia; 5 Psychology Department, Logan Hospital, Logan, QLD Australia; 6 Florey Institute of Neuroscience and Mental Health, Heidelberg, VIC Australia; 7 Rehabilitation Unit, Logan Hospital, Meadowbrook, QLD Australia

**Keywords:** Brain injury, Cognitive assessment, Classification, Immersive virtual reality, Unilateral visuospatial neglect

## Abstract

**Background:**

In neurorehabilitation, problems with visuospatial attention, including unilateral spatial neglect, are prevalent and routinely assessed by pen-and-paper tests, which are limited in accuracy and sensitivity. Immersive virtual reality (VR), which motivates a much wider (more intuitive) spatial behaviour, promises new futures for identifying visuospatial atypicality in multiple measures, which reflects cognitive and motor diversity across individuals with brain injuries.

**Methods:**

In this pilot study, we had 9 clinician controls (mean age 43 years; 4 males) and 13 neurorehabilitation inpatients (mean age 59 years; 9 males) recruited a mean of 41 days post-injury play a VR visual search game. Primary injuries included 7 stroke, 4 traumatic brain injury, 2 other acquired brain injury. Three patients were identified as having left sided neglect prior to taking part in the VR. Response accuracy, reaction time, and headset and controller raycast orientation quantified gameplay. Normative modelling identified the typical gameplay bounds, and visuospatial atypicality was defined as gameplay beyond these bounds.

**Results:**

The study found VR to be feasible, with only minor instances of motion sickness, positive user experiences, and satisfactory system usability. Crucially, the analytical method, which emphasized identifying 'visuospatial atypicality,' proved effective. Visuospatial atypicality was more commonly observed in patients compared to controls and was prevalent in both groups of patients—those with and without neglect.

**Conclusion:**

Our research indicates that normative modelling of VR gameplay is a promising tool for identifying visuospatial atypicality after acute brain injury. This approach holds potential for a detailed examination of neglect.

**Supplementary Information:**

The online version contains supplementary material available at 10.1186/s12984-023-01283-9.

## Background

Assessments of visuospatial attention problems following brain injury have focused on unilateral spatial neglect, which classically manifests as a bias toward ipsilesional space that renders objects in the contralesional space difficult to orient toward, perceive, and act upon [1–4]. Symptoms can manifest in activities of daily living, such as eating, when a person might eat food only from one side of their plate, and associate robustly and independently with poor prognosis, including long-term functional disability [5–9]. Although attention can recover during rehabilitation [10], current treatments produce uncertain evidence of efficacy [11, 12]. Neglect occurs most commonly following right-hemisphere stroke [13], but can also follow left-hemisphere stroke [14], traumatic brain injury (TBI) [15], and other causes of acquired brain injury (ABI) [16, 17]. Prevalence estimates of neglect vary widely (e.g., 24–68%) [18, 19], reflecting a variety of factors, including limitations of standard assessments [20].

In neurorehabilitation practice and research, pen-and-paper tests, such as clock drawing and letter cancellation, and behavioural tests routinely assess neglect [21–23]. While convenient, pen-and-paper tests have limited sensitivity, sometimes failing to detect even severe neglect symptoms [24, 25]. They also have variable specificity, for example, detecting problems with executive function rather than visuospatial attention [26, 27]. Many pen-and-paper tests do not assess severity or identify subtypes [22, 28]. For neglect assessment, computer-based methods provide alternatives. Among computer-based methods, immersive virtual reality (VR) appears ideally suited for neurorehabilitation and detailed neglect assessment [22, 29–33].

Immersive VR involves a headset (i.e., a head-mounted display) that simulates stereoscopic vision and allows the player to turn their head to explore three-dimensional (3D) computer-generated worlds [34]. VR hand controllers allow the player to select and interact with virtual objects requiring motoric coordination. VR uniquely and flexibly combines experimental control with sensory experiences that simulate naturalistic perception and motivate naturalistic behaviour [30]. This provides accessibility, the opportunity to individualise gameplay, and the capacity to map visuospatial attention with ecological validity (i.e., closer to a person's full field of vision as opposed to an A4 sheet of paper size).

VR is well suited to clinical settings as it is possible to manipulate the virtual world and build environments that might be unsafe for patients in the real world [35, 36, 49]. VR is also easily gamified therefore highly engaging [37, 38] facilitating participation in treatment [39]. The potential for VR to motivate patients in treatment, and for continuity in assessment and treatment approach, is another benefit to VR over current assessment approaches.

VR neglect tests have long been of interest and have been systematically reviewed and discussed [22, 33, 40–43]. However, the term “VR” has broadly included immersive VR [31, 44–57], precursor systems with stereoscopic shutter glasses [29, 58–61], headsets with incomplete degrees of freedom [62, 63], augmented reality systems [64–66], and 3D virtual environments presented on two-dimensional (2D) displays [54, 67–71]. There are also parallel computer-based neglect tests using 2D displays [24, 25], touch screens [28], and eye tracking [72–78]. Among these methods, immersive VR motivates behaviour most naturalistically while providing maximal sensory control.

In Table 1, we have summarised immersive VR neglect assessment studies [31, 44–57]. Collectively, these studies have shown that immersive VR manifests neglect symptoms in gameplay based on navigation, detection, obstacle avoidance, free viewing, street crossing, and cancellation. Additionally, these studies have shown that immersive VR can measure attention in ecologically valid contexts [49, 54, 55], correlate with neglect severity on standard tests [31, 50], detect mild neglect [51], distinguish between acute and chronic injuries [48], scale in difficulty for the individual [50], reflect behavioural responses to changing task demands [45, 55], measure orientation [47, 57] and heading direction [46], reveal the time course of recovery [51, 56], and reveal both spatial and non-spatial attention problems [53].

**Table 1:** Immersive VR neglect assessments

Study	Gameplay	Sample^a^	Neglect determination^b^	Feasibility^c^	Chronicity^d^ (M days)	Chronicity^d^ (SD days)	Case inferences
Current study	Localisation	Stroke, TBI, ABI, L/R/B	CLOX**, LCT**, LiCT**, observation*	GEQ, SUS, SSQ, chronicity	41	29	Normative modelling
Aravind et al. [38]	Navigation, avoidance	LHS, RHS	MVPT**, LCT**	None	413	706	Descriptives
Aravind and Lamontagne [39]	Navigation, avoidance	RHS	MVPT**, LCT**, BeT**	None	339	150	None
Aravind and Lamontagne [40]	Navigation, avoidance	RHS	MVPT**, LCT**, BeT**	None	339	148	Example results
Hougaard et al. [41]	Free viewing	RHS	KF-NAP^	None	84	103	Normative modelling
Jannink et al. [42]	Detection	LHS, RHS	BIT^	None	69	25	None
Kim et al. [43]	Street crossing	RHS	Deficits in ADLs^	None	116	97	None
Knobel et al. [30]	Cancellation	RHS	SNT**	SUS, SSQ	76	36	None
Knobel et al. [44]	Detection	RHS	CBS^, SNT**	SUS, SSQ, IPQ, PGTQ	64	33	Descriptives
Numao et al. [45]	Detection	RHS	Observation, LBT**, LiCT**, SCT**, CBS^	None	10		Case study
Ogourtsova et al. [46]	Navigation	RHS	LBT**, SCT**, AT**, observation*	None	675	589	Descriptives
Ogourtsova et al. [47]	Navigation, detection	RHS	LBT**, SCT**, AT**, observation*	None	675	589	Descriptives
Ogourtsova et al. [48]	Navigation, detection	RHS	LBT**, SCT**, AT**, observation*	None	675	589	Descriptives
Peskine et al. [49]	Navigation	RHS	BeT**, CBS^	None	490	865	Descriptives
Yasuda et al. [50]	Detection	RHS	Observation^, LiCT**, LBT**, CBS^	None	15		Case study
Yasuda et al. [51]	Detection	RHS	Observation^, TMT**, BIT**	Motion sickness	109		Case study

^a^LHS = left hemisphere stroke, RHS = right hemisphere stroke, L/R/B = left, right, and bilateral injuries

^b^** = pen-and-paper test, ^ = behavioral test, * = clinical observation, ADLs^ = Activities of daily living, Albert’s Test** = Albert’s Test, AT** = Apples Test, BIT^ = Behavioural Inattention Test, BeT** = Bells test, CBS† = Catherine Bergego Scale, CLOX** = CLOX: An Executive Clock Drawing Task, LBT** = Line Bisection Test, LC** = Letter Cancellation, LCT** = Letter Cancellation Test, LiCT** = Line Cancellation Test, KF-NAP^ = Kessler Foundation Neglect Assessment Process, MVPT** = Motor Free Visual Perceptual Test, SCT** = Star Cancellation Test, SNT** = Sensitive Neglect Test, TMT** = Trail Making Test

^c^GEQ** = Game Experience Questionnaire, IPQ** = Igroup Presence Questionnaire, PGTQ** = Perception of Game Training Questionnaire, SSQ** = Simulator Sickness Questionnaire, SUS** = System Usability Scale

^d^Time since injury

Immersive VR supports the notion that neglect affects both spatial and non-spatial attention [53]. A touchscreen neglect assessment recently extended this result and showed that neglect comprises subtype clusters defined by spatial and non-spatial effects [28]. Thus, neglect may not be unitary but reflect interactions between spatial and non-spatial attentional processes [28, 53, 79–81]. By extension, attention problems following brain injury may manifest distinctly across individuals in multidimensional trait space [82–84], defined here as individual-level behavioural performance patterns across a comprehensive suite of visuospatial attentional metrics.

Immersive VR neglect tests have progressed considerably, previous studies have several characteristics in common, as highlighted in Table 1. First, previous studies almost exclusively used right hemisphere stroke samples. While this approach provides scientific control, attention problems following left hemisphere stroke and ABIs were potentially missed. Second, previous studies determined neglect using existing assessments: pen-and-paper tests, behavioural tests, and clinical observation. These tests defined neglect and non-neglect groups, and thus the accuracy of existing pen-and-paper tests proceeded the accuracy of immersive VR. Third, previous studies focused on lateralised effects with an incomplete evaluation of non-lateralised effects. Fourth, most previous studies did not report feasibility, operationalised here as the acceptability of the VR gameplay and hardware. Further, most group studies recruited patients several months to years post-injury. Hence, feasibility for acute and subacute brain injury has not been broadly established. Fifth, although case studies and some group studies reported individual-level VR descriptive statistics, all but one study [47] did not report individual-level VR inferential statistics. This limits immersive VR for individualised rehabilitation.

To build on past work, we developed a self-referential immersive VR assessment of brain injury attention problems, including neglect. We achieved this via normative modelling, which provides individual-level statistical inferences based on expected patterns [47, 85–88]. We implemented normative modelling using a simple modification of outlier analysis. This leads us to introduce a new concept and mathematical property named “visuospatial atypicality”, which refers to whether the player’s performance on attentional metrics fell beyond outlier cut-offs based on patient and control groups. Visuospatial atypicality allowed us to examine attention problems across a variety of metrics that mapped flexibly onto individual-level patterns in multidimensional trait space.

This study was a pilot to examine the overall feasibility of VR, to prototype game levels, and to prototype the atypicality analysis among a broadly defined brain injury sample. The data were collected during heightened restrictions due to the global COVID pandemic and thus were challenging to obtain and of historical significance. The following results were based on the limited sample size and heterogeneous clinical characteristics of participants and are therefore preliminary. Therefore, the purpose of this report was to highlight the analysis methods, rather than the results. We recommend that future studies perform similar analyses with a larger sample size to establish atypicality cut-offs. Nonetheless, our new approach yields insights that can inform future extensive investigations.

## Methods

### Ethics approval

The current pilot study was part of a research protocol investigating the feasibility and validity of The Attention Atlas (AA) for neglect detection for inpatients with brain injury [32]. The study was approved by The Human Research Ethics Committees of Metro South Health (HREC/2021/QMS/70556) and Griffith University (GU Ref. No: 2021/179) and received Site-Specific Assessment authorisation from Metro South Health (SSA/2021/QMS/70556). All participants provided informed written voluntary consent consistent with the Declaration of Helsinki. The experiment was performed in accordance with relevant guidelines and regulations.

### Participants

We recruited clinician controls without brain injury and patients with brain injury from The Rehabilitation and Geriatrics Ward at Logan Hospital in Logan, Australia. Patients were eligible if they were clinically stable, had no history of epilepsy, had no reported visual field problems, had intact mobility of one or both hands, were not strongly susceptible to motion sickness (Simulator Sickness Questionnaire (SSQ) [89] scores < moderate nausea), and had high cognitive functioning on the orientation to time and space questions of Mini-Mental State Examination (scores ≥ 6 out of 10) [84]. Clinician controls were also required to be unsusceptible to motion sickness (SSQ scores < moderate nausea).

For patients and controls, we recorded age and sex. For patients, we additionally recorded time since injury, time in rehabilitation, diagnosis (stroke, TBI, and other ABIs), injured hemisphere (left, right, bilateral), and the Functional Independence Measure (FIM) [85]. The FIM is often routinely collected in Australian clinical practice and assesses independence on 18 activities of daily living on a 7-point scale (1 = total assistance, 2 = maximal assistance, 3 = moderate assistance, 4 = minimal assistance, 5 = supervision, 6 = modified independence, 7 = complete independence). The FIM is composed of two subscales, Motor and Cognition, with FIM Total calculated as the sum of the two. We expressed FIM on the original 7-point scale by dividing summary scores by the number of items (13 for Motor, 5 for Cognition, and 18 for Total).

### Procedure

A clinician team member examined medical files to assess patient eligibility, and eligible and interested patients provided verbal consent for academic team members to undertake recruitment. Following recruitment, an experienced neuropsychologist (author ML) administered three pen-and-paper tests of neglect to patients. Two days later, patients played VR for 19.5 to 28.7 min (see “Game levels”).

For the VR game, the experimenter first described immersive VR and the game and informed participants that there were free to discontinue at any time and for any reason. The experimenter fitted the VR headset. Participants first experienced the aurora night environment of Steam VR, with colourful auroras, mountains in the background, stars in the sky, and a ringed grid on the floor. After the game, participants reported their subjective experiences of VR on questionnaires assessing simulator sickness, game experience, and system usability. The experimenter also described the gameplay results, emphasising the developmental nature of the technology and analyses, describing areas of strengths, and avoiding phrases such as “attention deficit”, “spatial bias”, and “abnormality”.

### Pen-and-paper neglect tests

Three pen-and-paper tests presented a page at the patient’s midline. Patients had neglect if indicated on ≥ 1 test, and the neuropsychologist attributed the results to neglect rather than other causes. CLOX required patients to draw the numbers and hands on an analogue clock face to read legibly a time of 1:45. Once patients understood and drawing began, we provided no further help [95]. A preponderance of numbers on either side of the clock indicated neglect. The single letter cancellation test (SLCT) asked patients to cross out each letter “H” among an array of uppercase letters on a page. Four or more omissions on one half of the page compared with the other indicated neglect. Albert’s Test asked patients to cross out all the lines [87]. The neuropsychologist illustrated this by crossing out the five central lines. We encouraged patients to continue until they had crossed out all the lines. If > 70% of uncrossed lines were on one half of the page, neglect was indicated [88].

### Feasibility of The Attention Atlas

The SSQ [89] asked players whether they felt motion sickness during VR (1 = no symptoms, 2 = stomach awareness, 3 = mild nausea, 4 = moderate nausea, 5 = severe nausea, 6 = retching, 7 = vomiting). The game experience questionnaire revised (GEQ-R) [90, 91] asked players to indicate gameplay experiences on a five-point scale (1 = not at all, 2 = slightly, 3 = moderately, 4 = fairly, 5 = extremely) on 25 items, including “I felt content” and “I felt skilful”. Four factors of positive affect, competence, negativity, and flow were the mean of individual items. The SUS [92–94] asked players to rate VR hardware usability on 10 five-point (strongly disagree, disagree, neutral, agree, strongly agree) items, including “I think I would like to use this system frequently” and “I found the system unnecessarily complex”. Summed total scores categorised system usability as acceptable (70 to 100), marginal (50 to 70), or unacceptable (0 to 50).

### The Attention Atlas

#### Overview

**Fig. 1:**
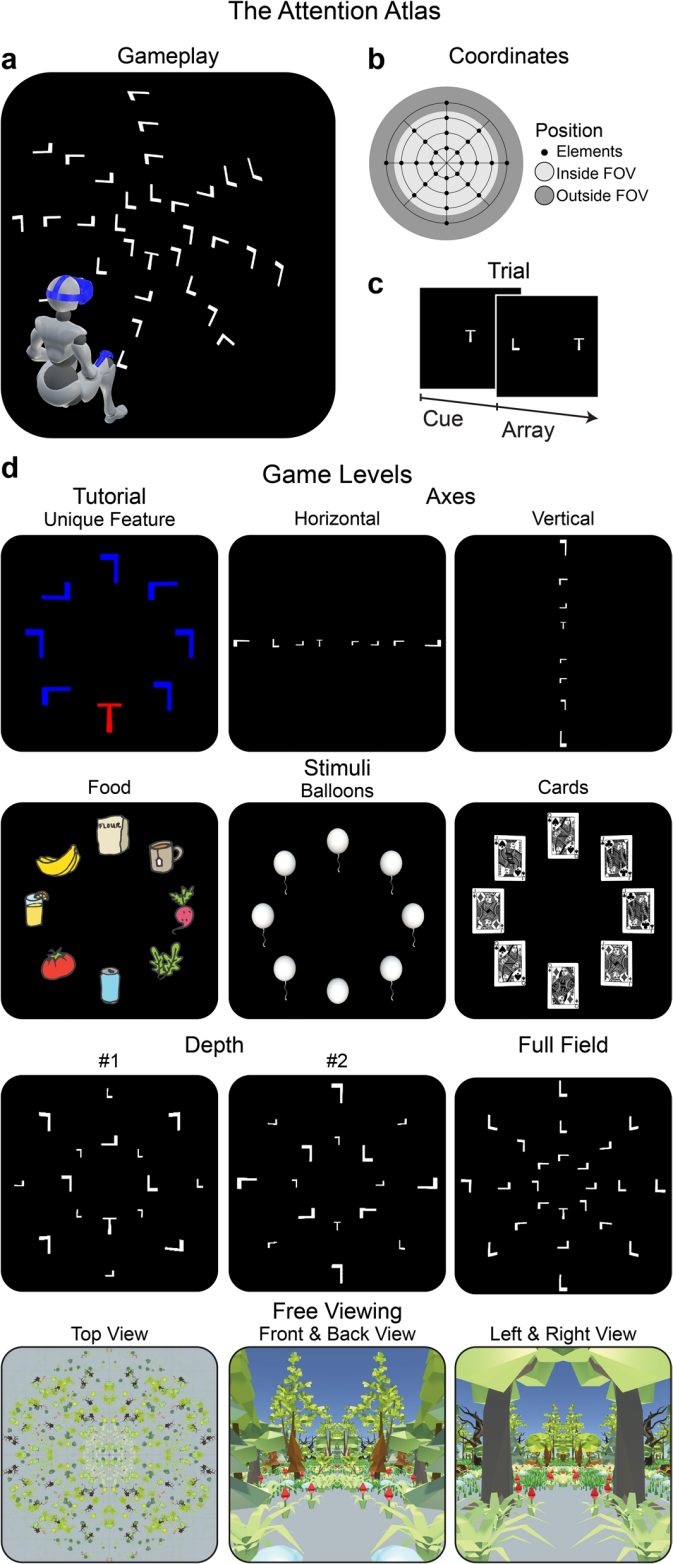
The Attention Atlas. **a** Visual search localisation gameplay. Array elements were positioned on a spherical surface with an origin at the headset position, calibrated at the start of each level [31]. This panel depicts the full field level. **b** Coordinates. Array elements were presented on a spherical grid. Each game level used a subset of possible positions, which could appear within the central field of view (FOV) or towards or beyond its edge, requiring head and eye movements for target localisation. For the axes level, the most eccentric horizontal and vertical positions fell outside the central FOV. **c** Cue/array trial structure. Cues and arrays were presented until an element was selected. **d** Game levels are depicted from the first-person perspective. Stimuli are scaled for clarity. Those with eight elements were presented on a single concentric ring presented 15° from central vision. The *tutorial*, excluded from analysis, was a search of a red “T” among blue “Ls”. *Axes* was a search for a “T” among “Ls” positioned horizontally or vertically on separate trials. *Stimuli* were food, cards, and balloons on separate trials. For food, the target and distractors are randomly selected at the beginning of each trial from 121 food icons. The queen of diamonds was the target for cards. For balloons, the target was a balloon without a string located among balloons with strings. *Depth* presented elements simultaneously at one of two depths: a near-surface (2 m) and a far-surface (4 m). *Full field* presented elements in four concentric rings. *Free viewing* depicted a low-resolution polygon forest, which surrounded the player in 360°. We instructed players to “look around” and report what they could see

We used The Attention Atlas [32] to assess visuospatial atypicality. The Attention Atlas has the player search for a single target among distractors (Fig. 1a) [94, 95]. Players sat in a non-swivelling chair or wheelchair. Players used their preferred hand or both hands for increased stability as preferred. Patients with upper limb paralysis used their more mobile hand. The experimenter sat or stood directly behind the player where possible and provided ongoing instructions, monitoring, and encouragement facilitated by the experimenter’s computer display. We used spherical coordinates (12.5° radial spacing, 15.0° concentric spacing, 2-m radius) to position search array elements (Fig. 1b) and an HTC Vive Pro headset (HTC, Taiwan) running on an Alienware Aurora R8 desktop computer (i7 9700, RTX 2070).

On each trial, a target cue appeared centrally. After the player selected the cue, the search array appeared, and the player located the target among distractors (Fig. 1c). The player selected the cue and target by pointing the VR hand controller and virtual laser pointer and pressing the thumb button. We instructed the player to perform as quickly and accurately as possible. If the player could not find the target, we instructed them to select one of the distractors. We did not assign such responses a unique error type. Without constraints, the player moved their head, eyes, and hand to locate and select the target. Correct target localisation was rewarded with a colourful confetti particle system instantiated at the target’s 3D coordinates. Distractor selection produced no confetti. The AA uses basic gamification features such as goals, rapid feedback and reinforcement, visible feedback [96–98], using levels (or an increase in difficulty), clear and simple game ‘rules’ [97, 98] and most importantly for patient engagement, fun and playfulness [97].

#### Game levels

Of the clinician controls, the first *n* = 4 undertook a longer game with eight levels. Based on their feedback, we reduced the total minimum level gameplay duration from 28.7 min to 19.5 min by dropping two levels and shortening others. Thus, the remaining controls (*n* = 5) and all patients (*N* = 13) undertook a shorter game with six levels (named tutorial, axes, stimuli, depth, full field, and free viewing). All levels lasted a fixed minimum duration and ended when an element (target or distractor) was selected. There was no fixed maximum duration (i.e., time-out). The level duration was proportional to the number of array elements multiplied by the number of stimulus combinations (tutorial, 0.5 min; axes, 3.0 min; stimuli, 4.5 min; depth 6.0 min; full field, 4.5 min; free viewing, 1.0 min). Variations of spatial extent and stimulus properties across game levels provided game progression (Fig. 1; see Additional file 1: Video S1 online—note, this is the clinician view, patients only saw the targets and distractors as in Fig. 1).

### Statistical analysis

#### Inferential statistics

We used non-parametric methods throughout to account for non-Gaussian distributions. The univariable and bivariable outlier analyses described below make no distributional assumptions.

#### Demographics

We compared FIM Motor and FIM Cognitive scores using Wilcoxon signed-rank tests.

#### Feasibility of The Attention Atlas

We compared the subjective experiences of the patient and control groups. For motion sickness, we used a χ^2^ goodness-of-fit test. For game experience and system usability, we used Wilcoxon ranked sum tests to assess midpoints and Levene’s test of homogeneity to assess variance.

#### Definition and summary of visuospatial atypicality

Visuospatial atypicality was a Boolean variable (0, 1) calculated for each attention metric and summarised across game levels as the mean, a Boolean proportion (0 to 1). We tabulated atypicality in a summary matrix (attention metric × player identifier [ID]). Player IDs were uniquely assigned and sequentially numbered according to the participation order. For each attention metric, we defined atypical gameplay as:

$$\begin{gathered} B_{{{\text{player}}}} \, = \,\left( {\left( {M_{{{\text{player}}}} \, < \,Q{1}_{{{\text{controls}}}} {-\!\!-}{1}.{5}\, \times \,IQR_{{{\text{controls}}}} } \right)|\left( {M_{{{\text{player}}}} \, > \,Q{3}_{{{\text{controls}}}} \, + \,{1}.{5}\, \times \,IQR_{{{\text{controls}}}} } \right)} \right) \hfill \\ {\text{and}} \hfill \\ \left( {\left( {M_{{{\text{player}}}} < Q{1}_{{{\text{patients}}}} - {1}.{5 } \times IQR_{{{\text{patients}}}} } \right)|\left( {M_{{{\text{player}}}} > Q{3}_{{{\text{patients}}}} + { 1}.{5 } \times IQR_{{{\text{patients}}}} } \right)} \right), \hfill \\ \end{gathered}$$

where *B*_player_ is the Boolean (0, 1) of whether the game was an outlier, *M*_player_ is the game measurement, *Q*1 is the first quartile, *Q*3 is the third quartile, and *IQR* is the inter-quartile range for the specific metric. We chose a Boolean representation for atypicality to standardize various measures onto a single scale, making it easier both to compare them and to visualize patterns of atypicality.

We compared atypicality prevalence between patient and control groups using a *χ*^2^ goodness-of-fit test, with typicality (typical, atypical) counts tabulated by group. Counts were derived by summing across players and metrics within each summary matrix.

#### Attention metrics

There were 14 attention metrics based on three primary categories: accuracy (%), RT (s), and raycasts (°) for the headset and controller. Accuracy, RT, headset latitude mean, headset longitude means, controller latitude means, and controller longitude means were computed for each game level. The headset and controller forward vectors cast rays to hit a spherical surface (2-m radius) positioned at the headset origin, calibrated before each game level [31]. Raycasts were converted from Cartesian (x, y, z) to spherical coordinates (latitude, longitude, 2-m radius).

Accuracy and RT were pooled across game levels to derive “spatial preference” difference scores (Δ% and Δs, respectively), contrasting four spatial quadrants (left, right, up, down) and three eccentricities (12.5°, 25.0°, 37.5°). We recombined the same trials separately for each contrast. We contrasted left and right (LR) quadrants, up and down (UD) quadrants, eccentricities of 12.5° and 25.0°, and eccentricities of 25.0° and 37.5°. For quadrants, we ordered subtraction terms separately for RT and accuracy, such that positive scores showed a spatial preference for right and up quadrants, and negative scores showed a spatial preference for left and down quadrants. For eccentricity, we ordered subtraction terms similarly for accuracy and RT. RTs were calculated for correct trials after individual-level outlier removal, performed separately for each level (level performance) or trials altogether (spatial preference). RTs outliers were defined by:

$$B_{{{\text{RT}}}} \, = \,\left( {\left( {T_{{{\text{RT}}}} \, < \,Q{1}_{{{\text{player}}}} {-\!\!-}{1}.{5}\, \times \,IQR_{{{\text{player}}}} } \right)|\left( {T_{{{\text{RT}}}} \, > \,Q{3}_{{{\text{player}}}} \, + \,{1}.{5}\, \times \,IQR_{{{\text{player}}}} } \right)} \right),$$

where *B*_RT_ is the Boolean of whether the trial RT was an outlier, *T*_RT_ is the trial RT measurement.

#### Data availability

All relevant anonymised and cleaned raw data and results are freely and publicly accessible on Open Science Framework (https://osf.io/staj7/) in Study1DataCleanCopy.zip (cleaned game IDs: study.Study1.gameLogMaster.feather; cleaned demographics: study.Study1.GUID.demographics.feather; cleaned feasibility questionnaires: study.Study1.questionnaires.feather; player summary results: PlayerSummary.py\; raw VR data: _RECORDINGS\_DATA; raw results: _RECORDINGS\_RESULTS\).

#### Code availability

Complete source code for the latest version of the analysis is available at (https://osf.io/staj7/; *AA.Diagnostics.zip*). Complete source code and most source files for the latest version of The Attention Atlas are available at (https://osf.io/staj7/; *AA.Standalone.zip*). LemonadePixel’s food illustrations (https://www.shutterstock.com/image-vector/hand-drawn-food-drink-icons-breakfast-716980507) and PolyWorks’ low-resolution polygon forest (https://assetstore.unity.com/packages/3d/environments/low-poly-forest-pack-polyworks-52733) are available in the build but not in the source files, since we do not own the distribution rights.

The games described in this report can be run from the presets (longer: *CliniciansTest1.game.json*; shorter: CliniciansTests2*.game.json*; demonstration: *CliniciansTestsDemo.game.json*) on the HTC Vive, HTC Vive Pro, and HTC Vive Pro Eye. Author DRP programmed the analyses in Python 3.10.6 and R 4.2.1 and The Attention Atlas in The Unity Game Engine 2019.4.20f1 on Windows 10.

## Results

### Demographics

Clinician controls (*N* = 9) completed all game levels (*axes*, *stimuli*, *depth*, *full field*, *free viewing*). Of the 18 patients recruited, two withdrew before VR, and one was excluded before VR due to susceptibility to motion sickness (SSQ score of vomiting). Incomplete or invalid data excluded two patients; the first patient discontinued during the second game level due to a lack of enjoyment; the second had unreported visual field loss. Therefore, the sample was *N* = 9 controls and *N* = 13 patients. Median ages were 59 years (IQR, 21 years) for patients and 43 years (IQR, 14 years) for controls. For patients, there were nine males, and for controls, there were four males.

Table 2 presents patient demographics. We recruited patients at a mean of 41 days (SD, 29 days, range, 17 to 125 days) post-injury, with patients having spent a mean of 14 days (SD, 14 days, range, 3 to 53 days) at inpatient hospital rehabilitation. The hospital diagnosed seven with stroke, four with TBI, and two with other ABI. Brain injuries were right-lateralised for six, left-lateralised for four, and bilateral for three. Patients ranged from complete independence to complete dependence on the functional independence measures (FIMs). Patients showed significantly greater cognitive (median, 5.6; IQR, 1.8) than motoric independence (median, 3.4; IQR, 3.3; *W* = 8.0, *P* < 0.001). Three patients (23.1%; patients 17, 19, 21) showed left-side neglect on ≥ 1 pen-and-paper test and the neuropsychologist agreed the result was due to neglect rather than other causes (identified in Table 2 as ‘neglect present’). One patient (patient 9) showed performance consistent with right-side neglect on the SLCT, but the neuropsychologist attributed this to causes other than neglect.

**Table 2:** Demographics of the 13 brain injury patients

Clinical characteristics										Neglect Pen-and-Paper tests				
Game ID	Age (Years)	Sex	Days since injury	Days in rehab	Diagnosis	Injured hemisphere	FIM motor^a^	FIM cognition^a^	FIM Total^a^	Neglect present (0/1)^b^	Neglected hemispace	CLOX (0/1)	SLCT (0/1)	Albert’s Test (0/1)
9	52	Male	53	6	TBI	Bilateral	3.4	6.2	4.2	0	Right	0	1	0
10	59	Male	52	4	TBI	Left	6.5	5.8	6.3	0		0	0	0
11	37	Male	37	7	TBI	Left	6.6	5.0	6.2	0		0	0	0
12	83	Male	17	7	Stroke	Left	6.0	6.2	5.9	0		0	0	0
13	65	Female	22	6	ABI	Right	5.0	5.6	5.1	0		0	0	0
14	80	Female	15	7	Stroke	Right	1.6	4.0	2.3	0		0	0	0
15	70	Male	23	14	ABI	Left	2.3	4.4	2.9	0		0	0	0
16	68	Male	125	9	TBI	Bilateral	1.2	2.2	1.5	0		0	0	0
17	47	Female	40	16	Stroke	Right	2.4	5.6	3.3	1	Left	1	1	1
18	47	Male	22	35	Stroke	Bilateral	5.3	7.0	5.8	0		0	0	0
19	66	Male	57	53	Stroke	Right	2.0	2.8	2.2	1	Left	1	1	0
20	48	Male	27	15	Stroke	Right	3.9	6.4	4.6	0		0	0	0
21	41	Female	42	3	Stroke	Right	1.5	5.4	2.6	1	Left	0	1	0

^a^We normalised FIM scores to the original 7-point scale (1 = total assistance, 2 = maximal assistance, 3 = moderate assistance, 4 = minimal assistance, 5 = supervision, 6 = modified independence, 7 = complete independence). ID = identifier

^b^As described in the methods, an experienced neuropsychologist determined neglect presence based on the results of three pen-and-paper tests. VR gameplay was not used for neglect determination

### Feasibility of The Attention Atlas

**Fig. 2:**
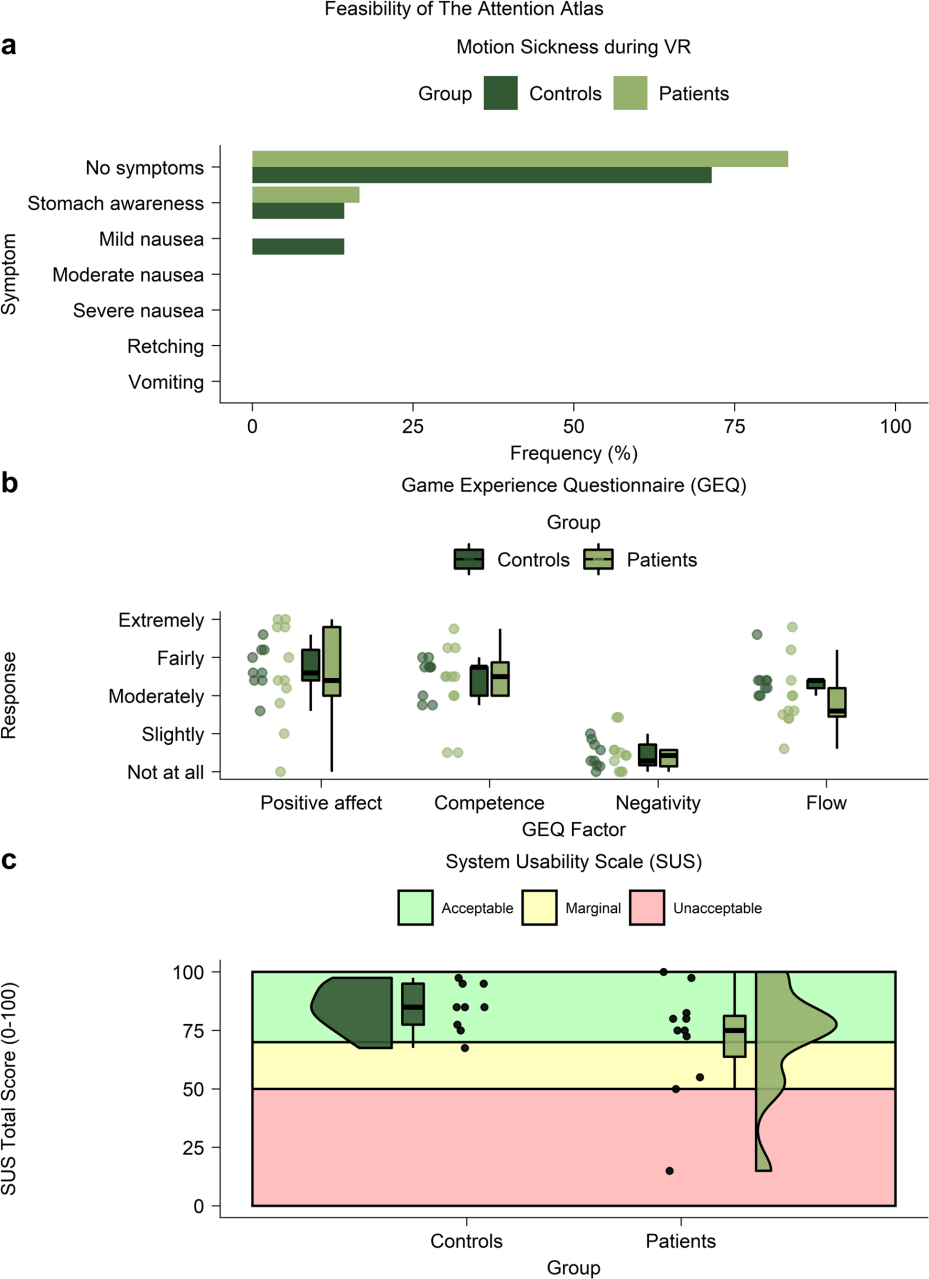
Feasibility of The Attention Atlas. **a** Motion sickness during VR on the SSQ. **b** Game experience on the GEQ-R (1 = not at all, 2 = slightly, 3 = moderately, 4 = fairly, 5 = extremely). This and subsequent box plots show the median, the 50th and 75th percentiles (at the upper/lower hinges), and 1.5 × IQR from the hinges (whiskers). In this and subsequent plots, points represent players. **c** System usability on the SUS. Shaded regions show acceptability cut-offs. This and subsequent half-violin plots show the kernel density estimation

We operationalised feasibility using subjective reports of the VR experience and present these results in Fig. 2. Because of the vagaries of the inpatient schedule, simulator sickness data were available for 10 patients and 7 controls, and game experience and system usability data were available for 9 controls and 9 patients. During VR, both groups reported no to minimal simulator sickness. Sickness severity did not differ significantly between groups (χ^2^ = 0.0, *P* = 1.0; Fig. 2a). The midpoint and variance of the game experience components of positive affect, competence, negativity, and flow did not differ between the groups (Wilcoxon ranked sum tests: *W*s < 1.9, *P*s > 0.06; Levene’s tests: *Ls* < 4.0, *P*s > 0.06). Overall, participants reported “moderate” positive affect (median, 3.6; IQR, 0.9), competence (median, 3.5; IQR, 0.8), and flow (median, 3.1; IQR, 0.8) and “not at all” negativity (median, 1.4; IQR, 0.4; Fig. 2b). Similarly, the midpoint and variance of system usability did not differ significantly between the groups (Wilcoxon ranked sum test: *W* = 1.4, *P* = 0.15; Levene’s test: *L* = 1.7, *P* > 0.21). The overall system usability was acceptable (median, 80; IQR, 13.1). System usability was unacceptable for one patient and marginal for two patients (Fig. 2c). Given minimal motion sickness scores, and positive results on the gaming experience, The Attention Atlas was feasible in our acute inpatient neurorehabilitation sample. However, it is worth noting 1 patient did not provide gaming experience data due to withdrawing because they did not enjoy the game. And although only 1 patient scored it as unacceptable, usability could also be improved.

### Summary of visuospatial atypicality

Normative modelling sought to detect visuospatial atypicality, defined as gameplay beyond the outlier cut-offs. There were 14 attention metrics based on three primary categories: accuracy (%), RT (s), and raycast orientation (°) for the headset and controller. Accuracy, RT, headset latitude mean, headset longitude means, controller latitude means, and controller longitude means were computed for each game level. Accuracy and RT were pooled across game levels to derive “spatial preference” difference scores (Δ% and Δs, respectively), contrasting four spatial quadrants (left, right, up, down) and three eccentricities (12.5°, 25.0°, 37.5°).

**Fig. 3:**
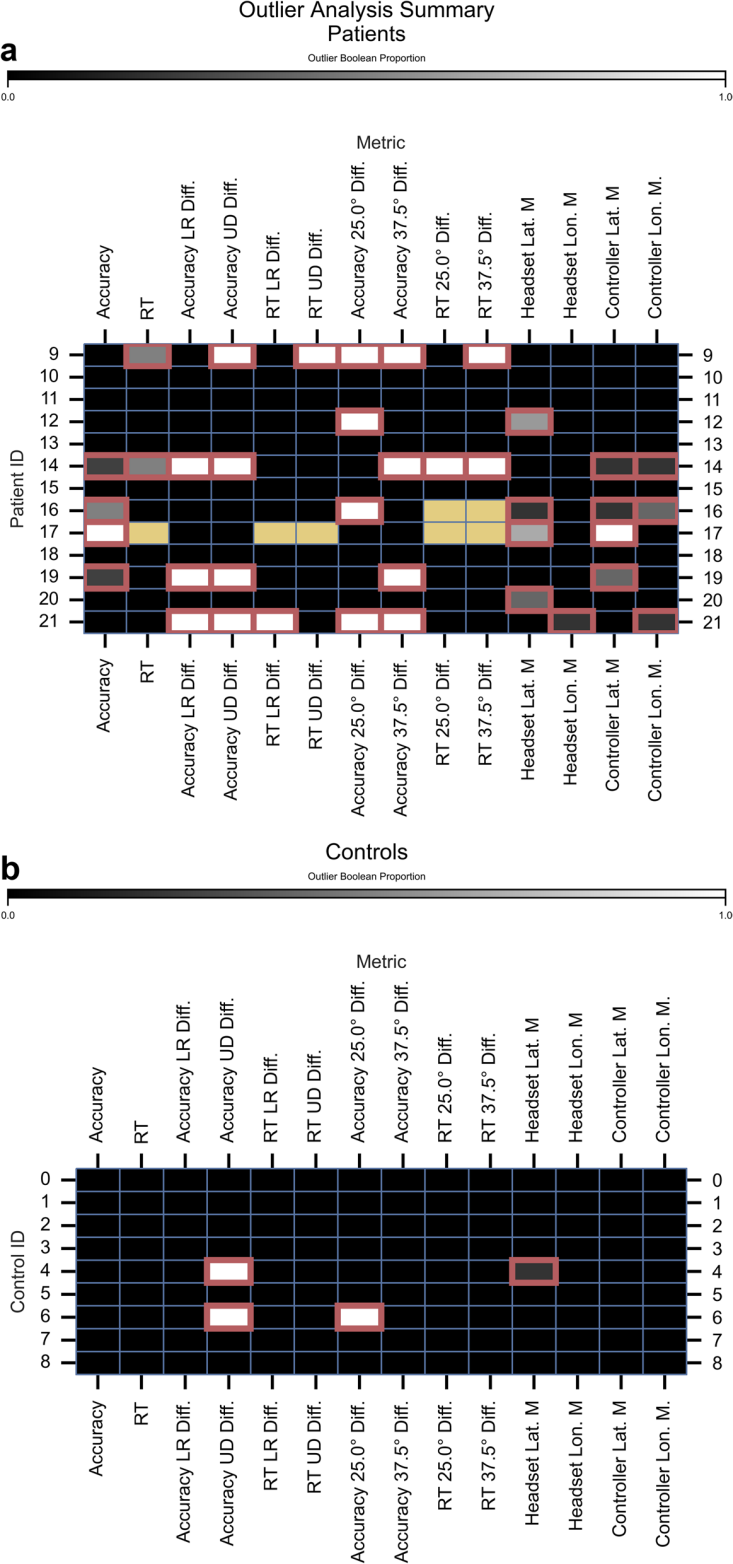
Summary of visuospatial atypicality. **a** Patient summary matrix. **b** Control summary matrix. The six primary performance metrics (response accuracy, RT, headset latitude mean, headset longitude mean, controller latitude mean, controller longitude mean) reflect the mean number of outliers across game levels. The vertical axis plots game ID, and the horizontal axis plots attention metrics. Black rectangles show outlier absence (i.e., typical performance), and non-black squares with red outliers show outlier presence (i.e., atypical performance). Yellow squares show missing data. For the first two metrics (accuracy and RT), outlier Boolean proportion (0–1) reflected the mean across game levels. LF = left/right contrast; UD = up/down contrast; 25.0° diff. = 12.5°/25.0° contrast; 37.5° diff. = 25.0°/37.5° contrast; lat. = latitude; lon. = longitude; M = mean

Figure 3 depicts the outlier summary matrices for patients and controls. These summary matrices showed that visuospatial atypicality was more prevalent in patients (Fig. 3a) than in controls (Fig. 3b; χ^2^ = 19.46, *P* < 0.001). For controls, 2 of 9 (22.2%) showed outliers, but only on one or two attentional metrics. For patients, 8 of 13 (61.5%) showed atypical visuospatial attentional patterns on ≥ 1 metric. For patients, case-wise, the number of atypical attentional metrics ranged from 0 to 9 of 14 (median = 2.0, IQR = 5.0). Further, combinations of outlier metrics differed across patients, suggesting heterogeneous atypical patterns.

**Fig. 4:**
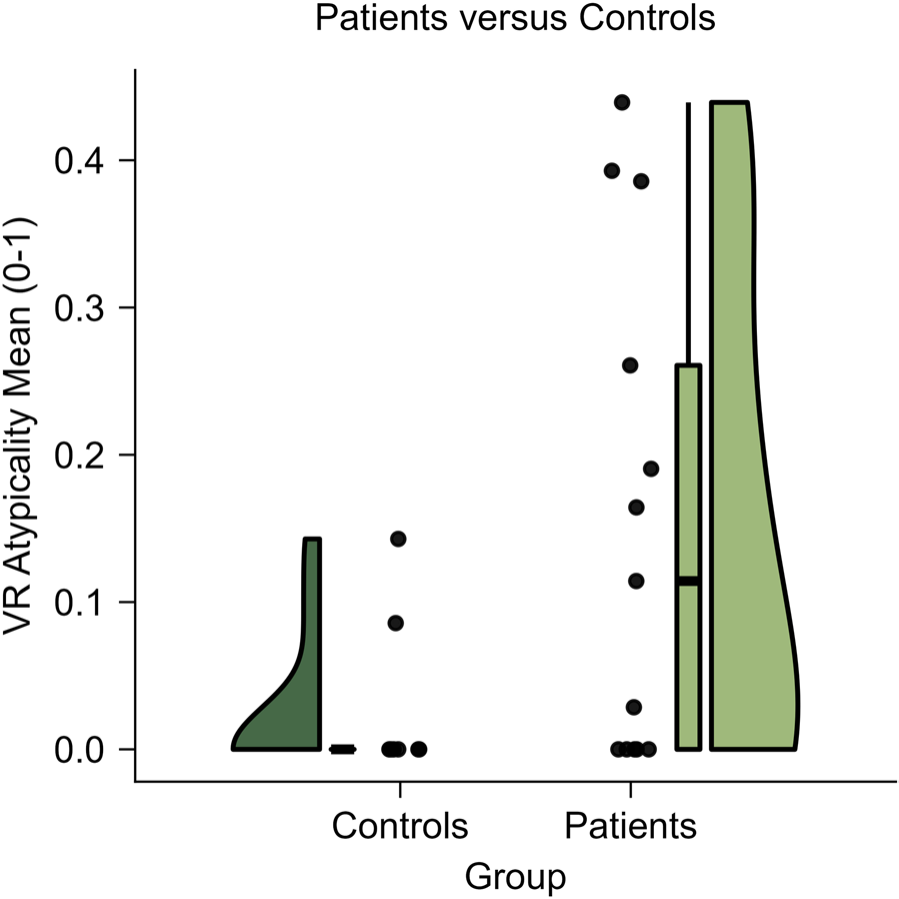
Mean visuospatial atypicality. Patients versus controls

To explore further, we calculated mean atypicality across all univariable metrics. Figure 4 depicts mean atypicality by group. The groups did not differ overall on mean atypicality (Wilcoxon ranked sum test: *W* = 1.9, *P* = 0.06) but differed in the variance of mean atypicality (Levene’s tests: *L* = 10.5, *P* = 0.004), with greater variance for patients (median, 0.1; IQR, 0.3) than controls (median, 0.0; IQR, 0.0). In summary, patients showed a greater range of atypical visuospatial patterns than controls.

### An illustrative case: using VR to elucidate unilateral spatial neglect in detail

Visuospatial atypicality described patterns specific to the individual. To illustrate this further, we will describe the gameplay of patient 17, who was among the most atypical individuals (mean atypicality = 0.19). Patient 17 was a 47-year-old female recruited 40 days post-injury, diagnosed with right hemisphere stroke, requiring maximal help with motor tasks, and showing modified independence with cognitive tasks, with left-side neglect on all three pen-and-paper tests. Indicated visuospatial atypicality constructs were attention challenge, headset orientation, and controller orientation. Patient 17 completed *axes*, *stimuli*, and *depth* game levels. *Full field* and *free viewing* level data were not available as the patient discontinued.

**Fig. 5:**
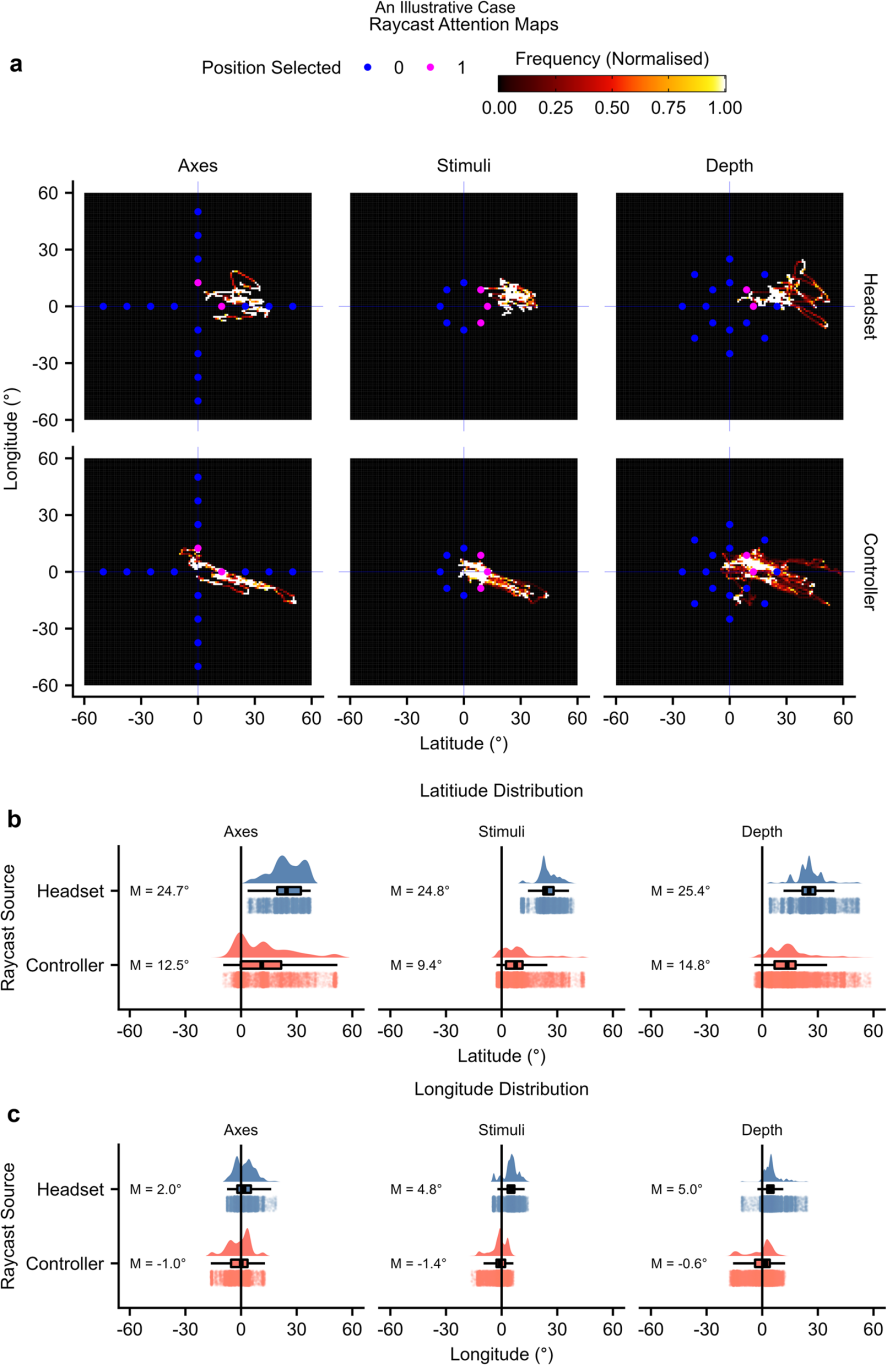
An illustrative case. This figure depicts raycasts for patient 17. **a** Headset and controller maps. For each level, longitude and latitude coordinates were converted into 2D histograms with bounds of -50° and + 50° and a bin width of 1°. Raycast maps were limited to search array rather than cue periods. Points represent array element locations. Blue points represent unselected locations, and pink points represent selected locations. **b-c** Raycast distributions. Boxplots, kernel density estimates, and individual frame measurements are depicted. **b** Raycast latitude means. **c** Raycast longitude means

Patient 17 completed 18 trials with an average RT of 51.4 s and did not find the target; hence, accuracy for all completed levels was atypically low (0%). The key result was a large spatial orientation and behavioural perseverance toward the right hemispace. Raycast hand controller data showed that the patient tended to select a distractor in the right hemispace (Fig. 5a). The headset and controller latitude means were atypically oriented rightward (9.4° to 25.4°; Fig. 5b). Thus, raycast attentional mapping showed orientational inattention to the contralesional hemispace. For axes and depth levels, many ipsilesional positions were not selected, suggesting additional inattention within ipsilesional space (Fig. 5a). Thus, using VR, we can delve deeper into analysing neglect compared to traditional pen-and-paper methods, examining multiple metrics such as RT, accuracy, and raycasts from various sources.

## Discussion

For attention problems following brain injury, we applied normative modelling of VR gameplay to characterise visuospatial atypicality, which referred to whether the individual fell beyond outlier cut-offs based on both patient and control data. The purpose of this paper was to pilot this approach, and to illustrate this with results, albeit with limited conclusions made on the efficacy of the AA based on these results. The results from this pilot showed that visuospatial atypicality was more prevalent among patients than controls. Atypicality corresponded highly but not entirely with pen-and-paper assessments. Visuospatial atypicality went beyond pen-and-paper, describing neglect in detail, describing attention problems on a variety of metrics, and detecting those missed or under-described on pen-and-paper. It is also worth noting that the frequency of outliers (i.e., potential neglect cases) found with the VR was what might be expected given findings in past literature [18, 19].

Pen-and-paper assessments are convenient and routinely used in neurorehabilitation practice and research [21, 22], including for evaluations of immersive VR neglect assessments. Thus, for integration into health systems, immersive VR must show feasibility. Here, we operationalised feasibility as the acceptability of the VR gameplay and hardware. For VR, the major known risk is simulator sickness, an experience like motion sickness, which is thought to arise through vestibular-visual mismatch [99, 100], although other factors can also contribute [101–103]. Such risks are minimised in modern low-latency systems and in experiences that exclude optic flow resulting from moving elements and the translation or rotation of the player’s perspective through space [104–106]. We verified that motion sickness was minimal for The Attention Atlas. Participants were monitored closely and encouraged to discontinue if they felt motion sickness or for any other reason. It is worth noting that we did exclude those who reported a history of motion sickness, so questions remain about this group. Of the patients recruited, only two discontinued – one because they did not enjoy the game and another who had unreported visual field deficits that made the game impossible [107–109]. Game experiences were overall positive, and system usability was acceptable or marginal for all but one patient. This suggests that the game is generally enjoyable, had a minimal chance of motion sickness, but that usability needs to be developed for patients to be able to fully engage with the AA. We conclude the AA was feasible, but more work needs to be done to explore motion sickness in higher risk patients, and more development to the usability of the game. Of previous immersive VR neglect assessments, only Knobel et al. [31, 50] investigated feasibility. Consistent with the current results, Knobel et al. found that VR produced minimal motion sickness in cancellation and detection games and positive game experiences and high system usability in a detection game. It is vital feasibility is measured in several ways for any new system being developed.

Consistent with feasibility, our sample comprised inpatients with stable cognitive function, who were recruited on a mean of 41 days (SD, 29) post-injury. Thus, VR was feasible within two months post-injury on average. Some previous immersive VR neglect test group studies recruited patients several months to years post-injury (e.g., [46, 53]), while others recruited patients two to three months post-injury [31, 47, 50]. Case studies have shown interpretable gameplay results within two weeks post-injury [51, 56]. Thus, the current study provides evidence for VR feasibility in groups of acute brain injury patients. This suggests that VR is feasible for acute and subacute rehabilitation and sensitive to acute attention problems, however, to validate feasibility in the groups included in this study (stroke, TBI, older/younger patients) larger samples of each will be required.

Consistent with the classical conception of neglect [1–4], visuospatial atypicality identified a spatial preference for and an orientation toward the ipsilesional space. Beyond this classical conception, the AA identified individuals with non-spatial atypicality who were typical spatially. This illustrates the potential for virtual reality and other computerised games to record multiple metrics and measure more than one construct.

The following section discuss the detection of spatial neglect using the AA vs pen-and-paper; however, these results are only preliminary, and should be interpreted as validating the analytical approach, rather than the efficacy of the AA to detect neglect. Visuospatial atypicality showed correspondence with pen-and-paper, detecting all three patients with left-side pen-and-paper neglect. Also, visuospatial atypicality identified three patients that pen-and-paper missed or under-described, with atypicality that could be consistent with spatial or non-spatial attention problems. Although a complete analysis of these patterns is beyond the current scope and statistical power of this paper, the results are broadly consistent with work showing strong positive correlations between VR performance and pen-and-paper metrics of neglect [30, 44] and neglect severity [49]. Note, though, that the current approach differs subtly but importantly from all previous immersive VR neglect assessment studies. Previous studies categorised patients as belonging to neglect and non-neglect groups based on existing tests and then stratified VR analyses accordingly. Thus, the VR results were dependent on the existing tests. By contrast, our VR analyses were self-referential, and our sample was recruited independently of existing tests and conceptualisations (i.e., we included stroke, TBI, and other ABI, and left, right, and bilateral injuries). It will be important for future work to examine the relationship between atypicality and real-world outcomes, such as prognosis and recovery.

Normative modelling of visuospatial atypicality quantified and conceptualised attention problems independently from current assessments. This approach has been used to chart brain morphology changes across the lifespan [80, 109], conceptualise the heterogeneity of various psychiatric disorders [79, 82], identify the consequences of stroke using smartphone interactions [110], and identify white matter anomalies in TBI [111]. In addition to independence from existing tests, normative modelling allowed individual-level statistical inferences, which facilitates potential for individualised rehabilitation.

Of the previous immersive VR studies, only one used normative modelling [41]. That study compared free viewing VR performance between controls and right hemisphere stroke patients with mild to severe neglect, determined via a sensitive behavioural test. The authors used 95^th^ percentile cut-offs to identify gaze and headset horizontal asymmetries based on control group data. The results showed atypical performance was detected more frequently in patients than controls and that 12 of 18 patients showed a rightward preference on one or more horizontal asymmetry metrics. Thus, within a neglect-defined sample, normative modelling can identify atypical gaze and headset neglect-consistent patterns.

The current study differs from Hougaard et al.’s work in three primary ways. First, our sample was defined by brain injury, and hence the VR analysis was independent of existing tests. Second, we used a localisation task rather than free viewing, which allowed RT and accuracy measurement and the quantification of non-spatial attention problems. Third, we used VR without eye tracking. The differences sum to a quantification of performance and orientation trait space following brain injury in the current study and quantification of horizontal asymmetries of orientation and gaze following right hemisphere stroke and neglect in Hougaard et al.’s work. This is an interpretation based on a small sample and larger studies are needed to explore this.

The results of Hougaard et al. and other eye-tracking studies [66–72] suggest that eye tracking can also be a sensitive measure. Converging evidence from multiple sources—performance, orientation, and gaze—may quantify diagnostic certainty for self-referential neglect/non-neglect classification. The trend toward eye tracking as standard in immersive VR headsets for interaction, diagnostics, and foveated rendering should facilitate this approach. Until eye tracking is widely accessible, assessment based on orientation and performance may have greater potential for immediate impact.

Normative modelling, concerning the current study, had several notable characteristics. First, the analysis relied only on broadly defined categories of patients and controls and therefore applied to heterogeneous samples, including various injury categories. Second, the analysis described diverse individual patterns, which were otherwise undetectable to pen-and-paper. Third, the analysis did not show that atypical patterns were necessarily pathological. Fourth, although the analysis weighted all metrics equivalently, all metrics might not have had the same functional significance. Fifth, the analysis did not categorise individuals as having neglect but identified visuospatial atypicality, which may be consistent with neglect. Sixth, the analysis depended on the sample such that the same individual might be typical or atypical, depending on the gameplay of others. Seventh, the analysis quantified atypicality and thus highlighted those who might most need help within the current caseload.

### Limitations

As a pilot study this paper has several limitations. The results from the AA are reported to support the main goal of exploring the feasibility of the analytical approach, but efficacy cannot be suggested until we complete larger trials. Secondly, the controls were not matched and were a convenience sample taken from clinicians within the hospital. Future studies would benefit from matched controls e.g., patients’ partners, but this was not possible for the current study, and was a limitation, amongst others, that are a result of COVID reduced accessibility.

This study prototyped VR measures that were independent of current assessments. Due to sample size limitations, we were unable to thoroughly assess the validity and therefore clinical relevance of visuospatial atypicality. Therefore, the functional limitations of the ‘atypicality’ label cannot be used for clinical decision making at this stage. Future validation studies will collect data to explore criterion validity and the correlation of ‘atypicality’ as described by the AA with existing indicators of spatial neglect such as traditional assessments and injury location. Approaches for assessing external validity include measuring correlations between functional disability and atypicality. We do not currently have data to explore whether the AA (and therefore VR more generally) is less susceptible to executive dysfunction than pen-paper assessments. Patient 17 provides a good example of how executive function might be less of a confounder in VR due to the availability of converging evidence across multiple sources. Patient 17 was unable to find any targets, so if we were relying on accuracy and reaction time, we would not be able to identify neglect via an orientation to one side or another, but as we are able to receive data from other sources (headset and controller orientation), it is still possible to make a judgement. In pen-paper tests, data is often only coming from one source (e.g., accuracy across spatial quadrants or the left–right axis—like ‘spatial preference’). However, this is something that needs to be explored.

## Conclusions

Neglect, as classically understood, manifests as spatial preference toward ipsilesional space that renders objects in contralesional space difficult to orient toward, detect, and therefore perceive. The current results show that brain injuries produce functionally heterogeneous visuospatial atypicality patterns across individuals that manifest distinctly across bivariable components and metric pairings. Brain injuries affected performance, orientation, attention challenges, spatial preference, or a combination of these. Visuospatial atypicality included lateralised neglect-consistent patterns and other non-spatial attention problems that were undetectable to pen-and-paper. To advance assessment and ultimately promote recovery, future studies might optimise and individualise the game modelling to create self-referential assessments that are accessible for acute injury and applicable throughout rehabilitation. Self-referential neglect/non-neglect classification might benefit from converging evidence across multiple source dimensions. Methods for establishing functional significance, including correspondence with prognosis and recovery, will be important to promote successful rehabilitation.

### Supplementary Information

**Additional file 1: Video S1.** A complete and abbreviated gameplay demonstration.This video demonstrates experienced healthy observer gameplay from a complete and abbreviated game containing all game levels and inter-level calibrations (total level-time duration of 3.9 minutes). Gameplay capture is from the first-person perspective. The information overlay was visible on the experiment computer display and not from within VR. We did not use audio affirmations, instructions, and sounds during this experiment. The depicted frame is from the *depth* level and shows the target successfully being located before selection. Smaller items are those that appear on the further depth surface. Apparent frame latency jitter, especially during the free viewing level, is due to capture and video compression and does not reflect the smooth, low-latency VR experience. This can be verified by the system performance information on the overlay, which shows a stable 90 Hz. We undertook the recording on a computer with a GTX 980 video card, showing reliable performance on older hardware. This recording used a swivel chair; hence, mobility during free viewing was higher in the demonstration than during the experiment.

## Data Availability

All relevant anonymised and cleaned raw data and results are freely and publicly accessible on Open Science Framework (https://osf.io/staj7/) in *Study1DataCleanCopy.zip* (cleaned game IDs: *study.Study1.gameLogMaster.feather*; cleaned demographics: *study.Study1.GUID.demographics.feather*; cleaned feasibility questionnaires: *study.Study1.questionnaires.feather*; player summary results: *PlayerSummary.py\*; raw VR data: *_RECORDINGS\_DATA*; raw results: *_RECORDINGS\_RESULTS\*). Complete source code for the latest version of the analysis is available at (https://osf.io/staj7/; *AA.Diagnostics.zip*). Complete source code and most source files for the latest version of The Attention Atlas are available at (https://osf.io/staj7/; *AA.Standalone.zip*). LemonadePixel’s food illustrations (https://www.shutterstock.com/image-vector/hand-drawn-food-drink-icons-breakfast-716980507) and PolyWorks’ low-resolution polygon forest (https://assetstore.unity.com/packages/3d/environments/low-poly-forest-pack-polyworks-52733) are available in the build but not in the source files, since we do not own the distribution rights. The games described in this report were tested and can be run without modification on the HTC Vive, HTC Vive Pro, and HTC Vive Pro Eye from the presets (longer: *CliniciansTest1.game.json*; shorter: CliniciansTests2*.game.json*; demonstration: *CliniciansTestsDemo.game.json*). Author DRP programmed the analyses in Python 3.10.6 and R 4.2.1 and The Attention Atlas in The Unity Game Engine 2019.4.20f1 on Windows 10.

## Abbreviations

ABI: Acquired brain injury
CLOX: An Executive Clock Drawing Task
FIM: Functional Independence Measure
GEQ-R: Game Experience Questionnaire Revised
ID: Identifier
IQR: Inter-quartile range
LR: Left-Right Quadrant
PCA: Principal components analysis
Q1: First quartile
Q3: Third quartile
RT: Reaction time
SSQ: Simulator Sickness Questionnaire
SLCT: Single Letter Cancellation Test
TBI: Traumatic brain injury
UD: Up-down quadrant
VR: Virtual reality
3D: Three-dimensional
2D: Two-dimensional
